# Electrohydrodynamic Vortex Imaging: A New Tool for Understanding Mass Transfer in Surface‐Based Biosensors

**DOI:** 10.1002/elps.8137

**Published:** 2025-05-10

**Authors:** Pauline Zimmer, Oleh Andreiev, Marion Costella, Emmanuelle Laurenceau, Jean‐François Bryche, Jean‐Pierre Cloarec, Michael Canva, Marie Frénéa‐Robin, Julien Marchalot

**Affiliations:** ^1^ Ecole Centrale de Lyon, INSA Lyon, CNRS Université Claude Bernard Lyon 1, CPE Lyon, INL, UMR5270 Ecully France; ^2^ Ecole Centrale de Lyon Université Claude Bernard Lyon 1, INSA Lyon, CNRS, Ampère UMR5005 Ecully France; ^3^ Laboratoire Nanotechnologies Nanosystèmes (LN2) ‐IRL3463, CNRS Université de Sherbrooke, INSA Lyon, École Centrale de Lyon, Université Grenoble Alpes Sherbrooke Québec Canada; ^4^ Institute Interdisciplinaire d′Innovation Technologique (3IT) Université de Sherbrooke, 3000 Boulevard de l'université Sherbrooke Québec Canada

**Keywords:** alternating current electroosmosis | dielectrophoresis | surface‐based biosensors | top‐bottom electrodes | vortices

## Abstract

Surface‐based biosensor performance is generally limited by mass transfer, especially when detecting low‐concentrated species. To address this, dielectrophoresis (DEP) and alternating current electroosmosis (ACEO) can be combined to enhance mass transfer, increasing the target concentration near the sensor. This article presents a method for real‐time direct imaging of electrohydrodynamic (EHD) effects on a microparticle suspension within a microfluidic chamber enclosed by two opposing electrodes. This top‐bottom configuration was poorly studied in the literature for ACEO. The system presented thereby allows measurements of fluid flow profiles perpendicular to the electrode surface. The velocity of fluorescent latex microsphere tracers was measured as a function of signal frequency, potential, and electrolyte conductivity. This setup enables direct observation of vortices and particle‐depleted areas, offering a valuable tool for selecting optimal input parameters—such as electric field, conductivity, and electrode dimensions—to efficiently concentrate microparticles near the sensor. Additionally, a numerical model developed in COMSOL and adapted for this top‐bottom configuration enhances understanding of key parameters influencing EHD phenomena.

## 1 Introduction

Biosensors that employ surface‐based detection methods are utilized for various applications in detecting biological substances. They usually rely on specific surfaces modified with biological recognition elements to capture and detect target analytes. They may employ various technologies for detection (surface plasmon resonance [SPR], quartz crystal microbalance, electrochemical sensing, impedimetric sensing, etc.). In surface‐based sensors, mass transfer limitations can affect the performance or functionality of the sensor [1]. For instance, passive bacteria detection at low concentrations requires long interaction times between the sample and the sensor surface to ensure sufficient target capture. This prolonged interaction is not compatible with real‐time monitoring applications, and the detection limit of the sensor may not be reached within a reasonable timeframe.

Therefore, strategies to overcome such limitations are required to ensure timely measurements of target analytes. For example, microfluidic channels can be integrated into the sensor design to optimize the flow of the sample over the sensor surface. These channels help to deliver the analyte efficiently to the sensing regions, reducing diffusion distances and partially overcoming some of the mass transfer limitations [2]. When subjected to an external magnetic field, magnetic fluids or nanoparticles can also be used to manipulate fluids or microparticles, thereby promoting mass transport or mixing [3]. Although magnetism‐based approaches are effective in specific applications, limitations arise depending on the properties of the target molecules or microparticles being manipulated, as a costly and time‐consuming labeling step is required. Alternating current (AC) electrokinetic approaches also offer valuable tools for enhancing mass transfer, microparticle manipulation, and fluid control in microscale systems [4]. In particular, electrothermal effects (ETEs) can induce thermal gradients within the fluid, leading to convection currents. These convective flows help in mixing and transporting substances within the fluid, facilitating faster mass transfer by promoting the movement of molecules or microparticles to the sensor surface and reaction sites [5]. However, ETE is not suitable for all types of samples or fluids, as biological materials are often sensitive to high temperatures. AC electroosmosis (ACEO) induces fluid flow within microchannels or capillaries when subjected to an alternating electric field. This electrokinetic phenomenon occurs due to the interaction between the electric field and the electrical double layer (EDL) at the solid–liquid interface [6]. It enhances mass transfer by generating convective flows that aid in rapidly mixing and transporting molecules within microfluidic devices. Dielectrophoresis (DEP) involves the movement of microparticles in a non‐uniform electric field. The microparticles experience a force depending on their polarizability, leading them to either move toward regions of high field strength (positive DEP or pDEP) or low field strength (negative DEP or nDEP). Thus, DEP also promotes enhanced mass transfer by allowing selective trapping and concentration of target analytes [7]. The combination of ACEO and DEP effects has proven efficient in capturing cells and microparticles, or molecules, in electrochemical [8, 9], SPR [10, 11, 12], or impedance‐based sensing approaches [13].

The electrodes used in DEP and ACEO experiments can be either coplanar (i.e., arranged on a single substrate) or three‐dimensional (3D) (i.e., arranged in a top‐bottom configuration), depending on the specific requirements of the experiment. Although coplanar parallel electrodes or interdigitated electrodes are widely used for ACEO [14, 15, 16, 17, 18], only few studies provide images of vortices generated in these configurations. Green et al. obtained such images with a horizontal objective and camera, but few details were given on the experimental setup and no experimental velocity map was provided [14]. Side‐view visualization within microfluidic devices can be obtained by using a prism setup [18], but this solution can be difficult to implement as the microchannel walls need to be perfectly smooth, and internal reflections caused by the air/channel/water refractive index transitions may impact image quality. The approach chosen here consists in simply tilting the electrode system by 90°, which allows the use of a standard inverted microscope equipped with a camera.

Other electrode configurations, such as asymmetric top‐bottom electrodes exist but are less well documented. They are used for particle manipulation [19, 20, 21] or micromixing [22]. However, to the best of our knowledge, no cross‐sectional images of vortices have been provided for such electrode configuration. Moreover, although some numerical simulations have been proposed for this 3D electrode design, they were based on the use of an analytical expression of the electroosmosis velocity [19], which was derived for parallel coplanar plates [6]. Here, we therefore propose to explore this asymmetric top‐bottom electrode configuration both experimentally and numerically, with the aim of studying the influence of key parameters—such as applied voltage, frequency, medium conductivity, and geometry—on vortex formation and dynamics. This configuration includes a microstructured network of parallel electrodes on one side of the chamber and a counter electrode on the opposite side, enabling volumetric mixing and particle transport.

This article presents experimental results of flow streamlines imaged in a plane normal to the electrode surface, explaining in detail the methodology for capturing and analyzing these images. The imaging approach presented here is versatile and can be applied to other electrode configurations for electrohydrodynamic (EHD) studies. To complement the experimental work, a numerical model developed in COMSOL is presented, which predicts fluid and particle motion under different conditions and geometries for the top‐bottom electrode configuration. This model provides valuable insights into the interplay between ACEO and DEP effects, offering a powerful tool for designing and optimizing biosensors.

Ultimately, the goal is to leverage this detailed experimental and numerical framework to develop a hybrid biosensor combining ACEO–DEP effects with enhanced SPR imaging for real‐time detection of bacteria in water. Furthermore, this methodology is adaptable to other surface‐based biosensors, provided that the sensing surface is microstructured to serve as an electrode for electric field generation.

## 2 Physical Phenomena

### 2.1 DEP

DEP is a phenomenon that occurs when a polarizable particle is subjected to a non‐uniform electric field, resulting in a dielectrophoretic force acting on the particle.

It is expressed as follows:

(1) $$F_{\mathrm{DEP}}=2\pi \varepsilon_{m}r^{3}Re\left[f_{cm}\left(\omega \right)\right]\nabla {\left|E\right|}^{2}$$

with $\varepsilon_{m}$ the electrolyte permittivity and ω=2πf, where f is the applied frequency and E is the amplitude (rms) of the electric field.

The Clausius–Mossotti factor, $f_{cm}$, is given by

(2) $$f_{cm}\;\left(\omega \right)=\frac{\varepsilon_{p}-\frac{\sigma_{p}}{\omega }j-\left(\varepsilon_{m}-\frac{\sigma_{m}}{\omega }j\right)}{\varepsilon_{p}-\frac{\sigma_{p}}{\omega }j+2\left(\varepsilon_{m}-\frac{\sigma_{m}}{\omega }j\right)}$$

with $\varepsilon_{p}$ the particle permittivity and j the imaginary unit.

To determine the direction of the force, we must consider the dielectric properties (conductivity and permittivity) of both the particle and the fluid, as well as the frequency of the electric field, which together dictate the sign of the real part of $f_{cm}$. If the particle is more polarizable than the fluid ($Re(f_{cm})\;>\;0$), it experiences a positive force that attracts it to the areas where the field lines are most concentrated. Conversely, if the particle is less polarizable, it is subjected to a negative force that repels it from these regions.

### 2.2 ACEO

The ACEO phenomenon is a physical mechanism that induces charge motion within an aqueous medium. When electrodes are immersed in an aqueous medium and a voltage is applied between them, the ions in the solution move toward the electrodes with opposite charges. When an alternating voltage is applied, the ions change direction in response to the polarization of the electrodes. The layer of ions on the surface of the electrode is known as the EDL. The model used to describe this double layer is the Gouy–Chapman–Stern model, which includes a dense layer of charges adsorbed on the surface, called the Stern layer, and a diffuse layer with mobile charges [23].

When charges accumulate at an electrode/electrolyte interface, the system behaves like a capacitor with a non‐uniform charge density. The potential decreases exponentially across the double layer with a characteristic length known as the Debye length, which corresponds to the thickness of the double layer and is expressed as follows:

(3) $$\;\kappa^{-1}=\sqrt{\frac{\varepsilon_{m}RT}{\;F^{2}\sum_{i}{z^{2}}_{i}c_{i}}}$$

with *R* the gas constant, *T* the absolute temperature in Kelvin, *F* the Faraday constant, and *z_i_ and c_i_*, respectively, the valency and the concentration of the ion i.

A tangential electric field component is required to drive the diffuse layer charges, thanks to the Coulomb force. These charges move along the slipping plane at the slip electroosmotic velocity $u_{t}$ given by the Helmholtz–Smoluchowski formula (see the following equation), carrying the fluid along with them through viscous drag [24]:

(4) $$\;u_{t}=-\frac{\varepsilon_{m}\zeta }{\eta }E_{t}$$

where η refers to the viscosity, $E_{t}$ denotes the tangential component of E field, and ζ is the electric potential at the boundary between the electrode surface and the surrounding fluid, measured at the slip plane, located just outside the Stern layer in the EDL [25, 26] and is referred to as the zeta potential.

This slip velocity induces recirculations—or vortices—in the fluid through viscous entrainment. Several parameters, such as the medium conductivity, the applied voltage, and the frequency of the alternating potential, influence vortex formation. The frequency dependence of the velocity is attributed to the frequency‐dependent behavior of the double‐layer impedance. At very low frequencies, sufficient time is available for the double layer to form during each half‐cycle of the applied potential, resulting in a higher double‐layer impedance. The tangential electric field in the double layer is small due to the associated voltage drop, which results in a small slip velocity. Conversely, at high frequencies, the double layer does not have enough time to form fully, leading to low double‐layer impedance. In this case, because the charge in the double layer is reduced, the zeta potential becomes smaller, resulting once again in a small slip velocity. At intermediate frequency, the product of the tangential electric field and the zeta potential at the interface is maximized, resulting in highest slip velocity [6, 20, 27]. The conductivity of the medium also influences ACEO behavior through its impact on the double layer. Specifically, conductivity determines how quickly ions respond to the electric field and accumulate near to the electrodes and charges the EDL. For very low conductivity media, there are few ions in the bulk, requiring them to migrate long distances to balance the charges of the electrodes. As a result, the double layer takes longer to form. When the EDL is not fully formed, the electroosmotic flux is reduced. Conversely, higher ion concentrations in the fluid lead to a more compact double layer, formed quickly, and an enhanced screening effect, which reduces the zeta potential and decreases the electroosmotic velocity. The optimal frequency for ACEO flow also depends on the rate at which the AC field oscillates relative to the double‐layer charging time. In low‐conductivity media, the charging time is longer, requiring a lower AC frequency for optimal ACEO performance. High‐conductivity media allow for rapid EDL formation and require higher frequencies to prevent parasitic phenomena such as electrolysis or diffusion [27].

## 3 Materials and Methods

### 3.1 Electrode Fabrication

The electrodes used to generate vortices via ACEO consist of a plain gold electrode and a gold microstructured electrode fabricated using photolithography and wet etching techniques. These electrodes are arranged in a top‐bottom configuration at the front and rear of the microfluidic chamber, separated by a distance of 160 µm. The chamber is enclosed by double‐sided adhesive on the top and edges, with a microscope glass slide forming the bottom.

The microstructured electrode is comb‐shaped with finger widths of 100 µm and gaps of 200 µm. A 40 × 25 × 1.1 mm^3^ glass slide was first coated with a 50 nm layer of chromium followed by a 150 nm layer of gold (Neyco). The slide was then spin‐coated with AZ 5214 E photoresist (Microchemicals) at 3500 rpm for 30 s and post‐baked at 110°C for 1 min and 30 s.

Next, the sample was exposed to UV light at 365 nm using a UV LED‐based exposure system (KLOE, UV‐KUB) through an acetate photomask. The UV‐activated photoresist was developed using AZ 726MIF developer (Merck), following the supplier's protocol. The unprotected portions of the metal layer were etched using gold and chromium etchants (Merck) to produce the desired comb pattern. Finally, the remaining photoresist was removed with acetone.

### 3.2 Device Fabrication

**FIGURE 1:**
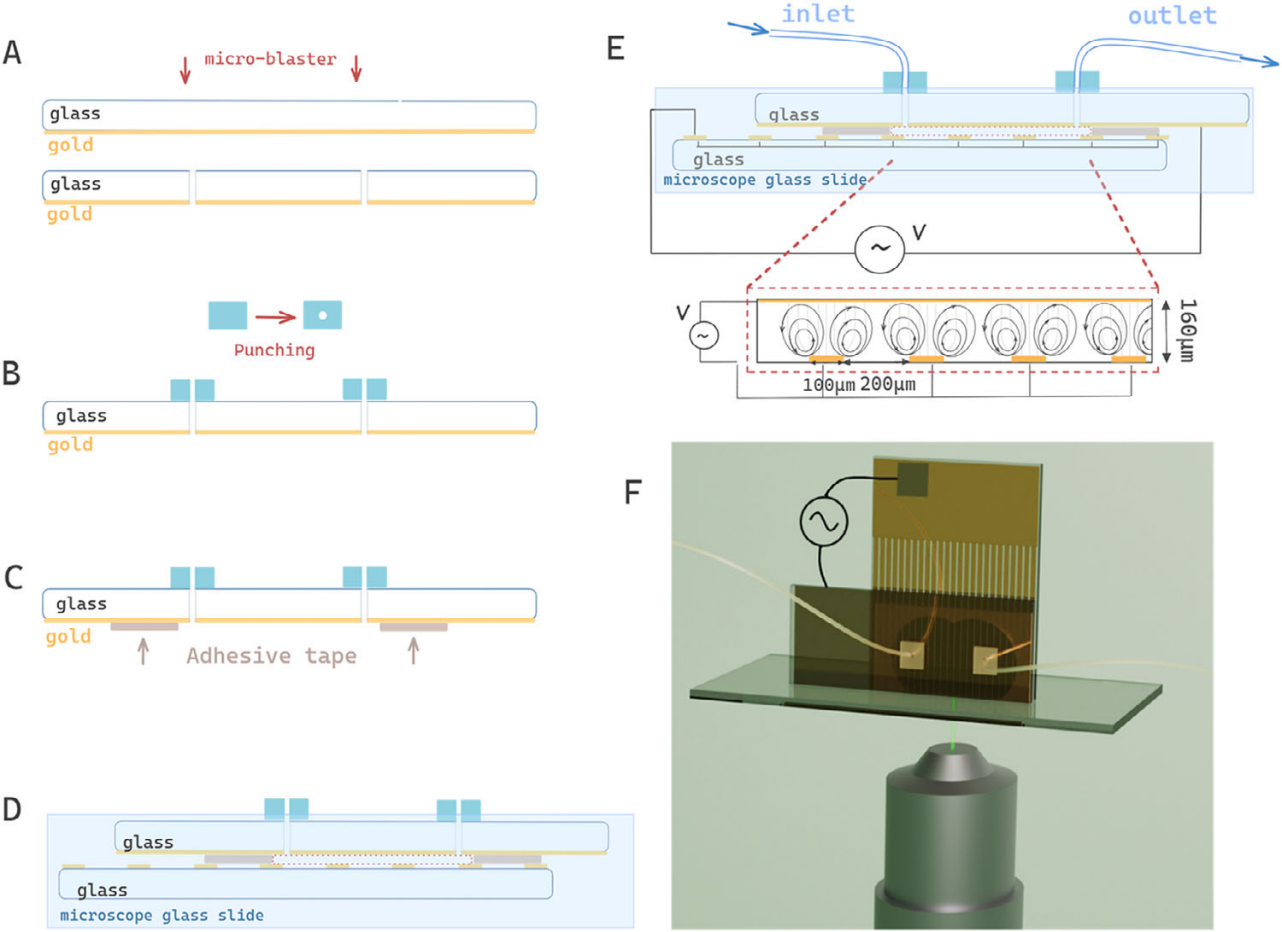
(A–E) Step‐by‐step creation of the device (bottom view); (E) zoom on the microfluidic chamber when the voltage is applied; (F) 3D rendering of the final device (front view).

The two electrodes described earlier are required to assemble the 3D electrode system. First, the gold plain electrode is perforated using a microblaster (COMCO, Burbank, California) to create two holes (Figure 1A). Next, two perforated pieces of polydimethylsiloxane (PDMS) (punched with a 1 mm tool) and the glass side of the gold plain electrode are treated with an air plasma cleaner for 1 min (Harrick Plasma Cleaner). This treatment ensures the permanent bonding of PDMS to the glass, forming the inlet and outlet of the microfluidic chamber (Figure 1B).

The microfluidic chamber, highlighted by red dotted lines in Figure 1E, is constructed using two layers of double‐sided adhesive stickers (ISpacer from SUNJin Lab, Hsinchu, Taiwan), each with a thickness of 80 µm, as measured by a Dektak surface Profiler (Veeco). The chamber design was cut from the sticker using a precision cutting machine (CAMEO, Silhouette), as illustrated in Figure S1. The two layers of ISpacer were then carefully placed onto the gold surface of the plain electrode (Figure 1C).

To complete the assembly, the two electrodes, bonded with adhesive ISpacer as shown in Figure 1D, were arranged vertically and secured to a microscope glass slide using UV glue (LOCTITE AA 3491, purchased from Farnell). Magnets or squares can be used to ensure that the electrodes are perpendicular to the glass slide. UV liquid adhesive, for sealing, was applied along the contact between the electrode and the glass slide, and curing was initiated on demand with a UV light source (SV43, Alonefire, England). Immediately after the adhesive was applied, it was exposed to UV light for at least 30 s (depending on the adhesive thickness) to ensure it cured before spreading into the chamber. The same process was repeated for the second electrode‐to‐glass slide contact, and an additional layer of glue was applied around the electrodes to ensure a proper seal.

Finally, the inlet and outlet tubing were connected to the microfluidic chamber, and the two electrodes were connected to a voltage generator (Agilent 33250A) using alligator clips (Figure 1E,F). To prevent contamination from dust, the electrodes and the glass slide were cleaned before assembly with acetone, ethanol, and deionized (DI) water and then dried with N2. Furthermore, all solutions introduced into the device were filtered through a 0.22 µm syringe filter before adding the 1 µm fluorescent microparticles.

### 3.3 Experimental Setup

Circulating flow was visualized using 1 µm fluorescent microbeads (Fluoresbrite Multifluorescent Microspheres, Polysciences, Bergstrasse, Germany). The beads were suspended in a buffer circulating within the system, which was controlled by a microfluidic flow controller (OB1, ELVEFLOW, Paris, France), as shown in Figure S2. Observations were performed using a Carl Zeiss Axiovert inverted epifluorescent microscope equipped with a Cy3 filter set and a 10× EC Plan Neofluar objective lens (Carl ZEISS). Movies capturing the behavior of microparticles under ACEO/DEP forces were recorded using a Photron Fastcam series high‐speed camera (Photron, Wycombe, UK) and analyzed with Photron Fastcam Viewer (PFV3) software.

An AC potential ranging from 4 to 8 V_pp_ at 1 kHz was applied to generate electrokinetic forces. To prevent loading effects, an amplifier with near‐zero output impedance (HSA4011, NF) was placed between the generator and the device.

### 3.4 Sample Preparation

Samples were prepared using DI water, fluorescent 1 µm microbeads, and potassium chloride (KCl) at varying concentrations to achieve conductivities ranging from *σ* = 0.5 mS/m to 30 mS/m. Conductivity was measured using a Mettler Toledo conductimeter equipped with an InLab 741 conductivity probe (Mettler Toledo, Viroflay, France).

The microbeads were initially rinsed with DI water and then resuspended in the KCl solution at the desired conductivity. The final concentration of microbeads was adjusted to 4.55 × 10^7^ particles/mL (experimentally determined to ensure a sufficient number of tracers for reliable velocity measurements).

### 3.5 Measurements and Image Processing

For each buffer conductivity, three measurements were taken at three different voltages (4, 6, and 8 V_pp_), with a frequency of 1 kHz. Each measurement consisted of a 10‐s video followed by an image capture 30 s after the recording. The recorded movie was subsequently processed using ImageJ.

Between conductivity changes, the device was rinsed first with filtered ethanol and then with filtered DI water. To detach the beads from the electrodes and restore a uniform particle distribution within the chamber, a back‐and‐forth flow was applied, ensuring consistent conditions (conductivity and frequency) between measurements while varying the voltage.

To avoid forming air bubbles that could disrupt the flow, particular attention was given to the chamber design, flow rate, and choice of liquid. The chamber was constructed without corners, and the flow rate was kept low when using water. Ethanol was selected due to its low surface tension, which allowed for efficient wetting of the chamber before flushing with water, helping to eliminate air bubbles.

A cleaning protocol was established to remove fluorescent microparticles that have adsorbed onto the electrodes after a series of observations and make the device reusable. A 2% solution of Hellmanex III (Merck) in DI water was injected into the device, which was then placed in an ultrasonic bath for 30 min at 40°C. The cleaning solution was subsequently removed by flushing the device with DI water, allowing the device to be reused.

The experimental image stack was processed using ImageJ, and the velocity was calculated by applying tracking commands from the TrackMate Plugin within the area of interest [28]. Vortices were visualized by overlaying all the images into a single one.

### 3.6 COMSOL Simulations

Numerical simulations were performed to model the effects of DEP and ACEO using COMSOL Multiphysics software. The goal was to simulate particle behavior in the microfluidic chamber under varying conditions, including changes to the chamber geometry.

The following study neglects the occurrence of faradaic reactions (such as electrolysis) and thus assumes ideally polarizable electrodes. Moreover, for simplicity, we assume that the double‐layer voltage is on the order of the thermal voltage, which allows us to treat the system of equations as linear [29].

The 2‐D geometric model shown in Figure S3 consists of a rectangle, the height of which corresponds to that of the fluidic chamber. The bottom gold electrode is represented by a polarizable segment 100 µm long, whereas the top electrode is modeled as a polarizable infinite segment.

Of note, many studies rely on the use of an analytical expression of the electroosmosis velocity for calculation [19, 30]. However, it is not applicable to all device geometries. Therefore, we used the numerical approach proposed by Green et al. instead [14].

The potential across the chamber was calculated by solving Laplace's equation.

$\nabla^{2}\phi =0$ using the following equation as a Robin‐type boundary condition on the electrode in COMSOL Laplace equation module:

(5) $$\sigma \;\frac{\partial \phi }{\partial y}=j\omega C_{\mathrm{DL}}\left(\phi -V_{i}\right)$$

where ϕ is the potential at any given location, $V_{j}$ is the potential applied to the electrode, $C_{\mathrm{DL}}$ is the double layer capacitance per unit area, and σ is the electrolyte conductivity.

This method provided the potential at the outer edge of the double layer, from which the slip electro‐osmotic velocity can be derived using Equation (6).

The time‐average slip velocity can then be expressed as follows [14, 17]:

(6) $$\langle u\rangle =-\frac{\varepsilon_{m}}{4\eta }\Lambda \frac{\partial }{\partial x}{\left|\phi -V_{i}\right|}^{2}$$

where *Λ* is the ratio between the double‐layer capacitance and that of the diffuse layer only, expressed as *Λ* $=\frac{C_{s}}{C_{d}+C_{s}}$.

This velocity was then imposed as a boundary condition at the electrode surface to estimate fluid motion by solving Navier–Stokes equations using COMSOL Laminar flow module, assuming that the fluid was incompressible. In this module, the following wall conditions were set: A non‐slip condition was applied on the upper and lower walls, except on the electrodes, where the slip velocity was defined by Equation (6), as illustrated in Figure S3.

A symmetry boundary condition was then applied to the right and left walls of the microfluidic chamber in the laminar flow module. A point constraint of zero pressure was also added to the four corners of the microfluidic chamber.

This method, which involves first solving the Laplace equation on the electrodes and then deducing the slip velocity, has the advantage of being adaptable to any geometry.

To track particle trajectories and count those captured on the electrodes, we used the “Particle Tracing for Fluid Flow” module. In this module, the behavior of a particle when it encounters a boundary needs to be defined. A “stick” boundary condition has been implemented to ensure that once a particle hits the surface, it remains attached. Indeed, because we are interested in counting particles that come into contact with the electrode surface, this condition allows particles to remain on it. Moreover, this choice aligns with the expected behavior of the target species, which should bind to the chemically functionalized sensor surface. The particles were subjected to drag (based on the velocity obtained from the laminar flow module) and DEP forces, which are both implemented in COMSOL. This particle tracking enabled the investigation of how chamber geometry influences microparticle capture. The properties of the microparticles (latex beads) are provided in Table S1.

## 4 Results and Discussion

### 4.1 Forces Involved

A dedicated observation system, combined with COMSOL simulations, provides valuable tools for investigating EHD effects on particles and understanding the interplay between DEP and ACEO‐induced flow velocity profiles.

When the 1 µm particles used in our study are subjected to an AC electric field with a frequency of 1 kHz, the dielectrophoretic force is theoretically found to be positive for medium conductivities lower than 4 mS/m (see Supporting Information section). The ACEO phenomenon, although not a force itself, induces vortices that mix the fluid throughout the chamber. This movement, driven by the drag force (see Supporting Information section), contributes to particle motion.

**FIGURE 2:**
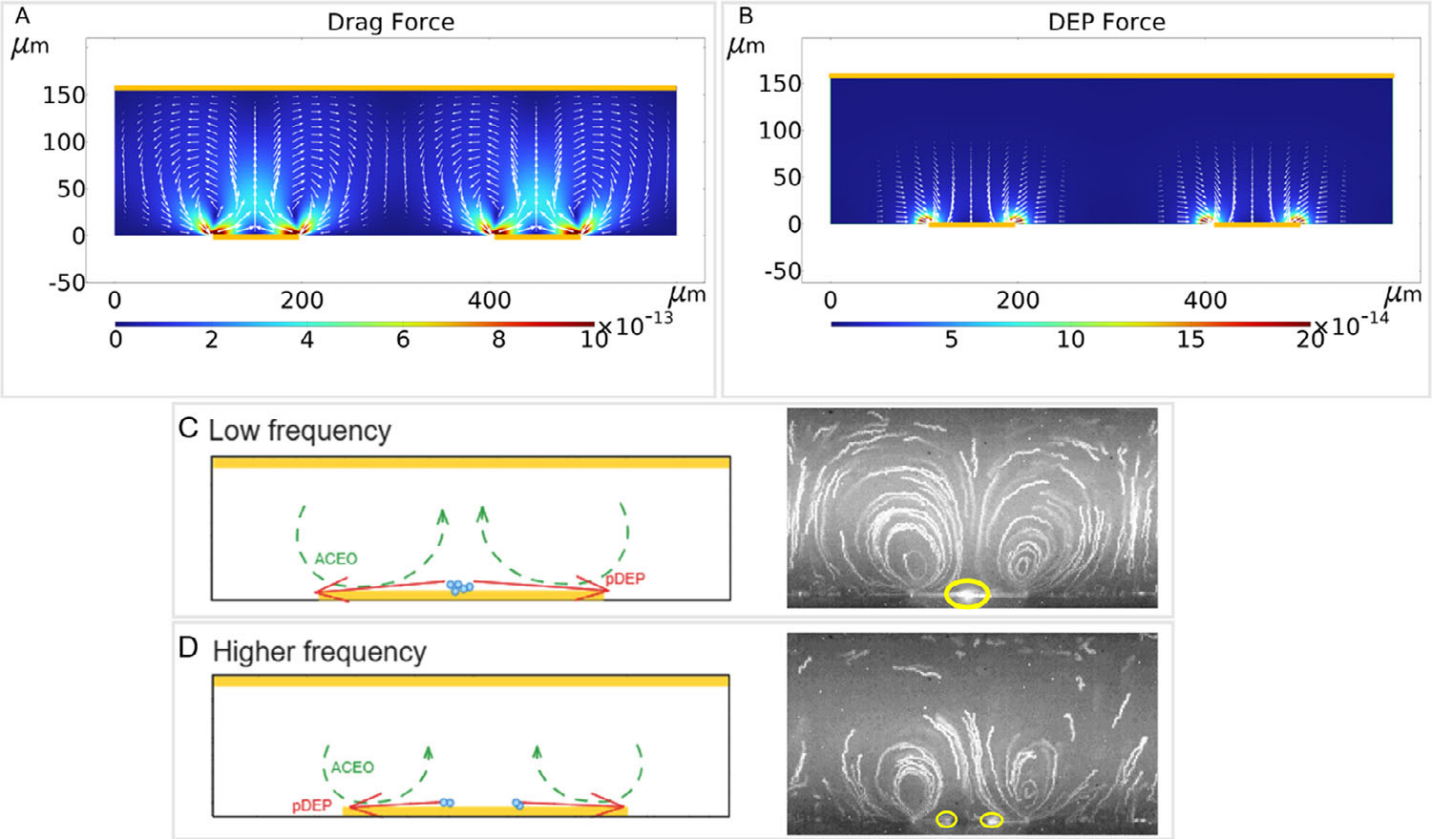
(A) Drag force and (B) DEP force: Force maps plotted on COMSOL with arrows representing the direction and magnitude of the force vectors. Parameters are fixed at 1 kHz frequency, 1 mS/m conductivity, and 6 V_pp_ voltage. Effects of ACEO and DEP on particle trapping under different electrokinetic regimes with images of vortices in which the areas of particle capture are circled in yellow at (C) low frequency (typically 1 kHz in the image at right) and (D) higher frequency (typically 2 kHz in the image at right). DEP, dielectrophoresis.

Although the DEP force attracts particles to regions of strong electric fields—specifically, the chamber edges, as shown in Figure 2B—the drag force propels the particles in circular trajectories, as illustrated in Figure 2A. Under the described conditions, ACEO effects dominate over DEP [19, 30].

As particles approach the electrode edges, they are accelerated by the pDEP force, which modifies the ACEO‐induced particle trajectories. This interaction causes particles to move toward a stable region near the electrode center, depending on the balance of forces. At these stable positions, the viscous drag force and the DEP force counteract each other, as depicted in Figure 2C,D. The precise location of these stable positions is influenced by experimental conditions, including frequency, medium conductivity, and applied voltage, all of which determine the balance between drag and DEP forces [11, 31].

### 4.2 Vortices’ Observation

Both experimental and numerical observations of vortices were performed using the device described in Section 3.2 and the COMSOL simulations outlined in Section 3.6.

**FIGURE 3:**
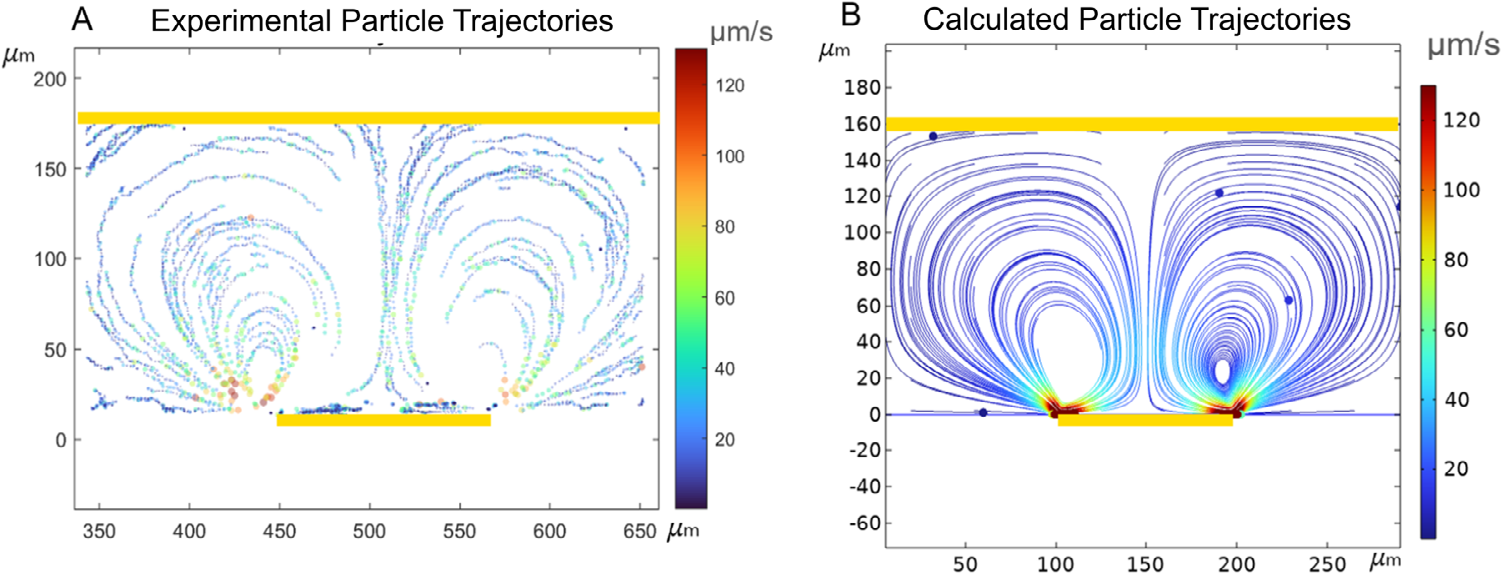
(A) Experimental particle trajectories obtained with image analysis are processed with MATLAB; (B) calculated particle trajectories plotted in COMSOL are corrected with a correction factor $\Lambda_{\exp}=0.02$. The yellow bars represent the gold electrodes. These two particle trajectories were obtained using these parameters: *V* = 6 V_pp_, *f* = 1 kHz, $\;\sigma_{m}=1\;\mathrm{mS}/m$.

The experimental velocity was extracted using tracking commands in ImageJ (TrackMate Plugin) within the area of interest [28]. The resulting data were then processed in MATLAB to generate a velocity map, as shown in Figure 3A. A corresponding numerical velocity map, obtained from COMSOL simulation, is represented in Figure 3B. Because numerical simulation typically overestimates fluid velocities compared to experimental results due to nonlinear dynamics in response to large voltage, a correction factor—$\Lambda_{exp}$—introduced by Green et al. [14, 16] and discussed by Bazant et al. [29, 32] was applied to the Helmholtz–Smoluchowski slip velocity (Equation 6) in the numerical simulation as a fitting parameter. It was determined by comparing experimental and numerical results, particularly in the formation of the depletion zone and the velocity of microparticles, so that the numerical results match the experiments. A correction factor between 0.016 and 0.025 enabled numerical results to align with the experimental data. It was set to 0.02 in COMSOL simulation to obtain the velocity map in Figure 3B, in the Supporting Information video, and the study of the influence of the chamber geometry shown in Figure 5. The low value of the correction factor is explained by the fact that relatively high voltages were applied to the double layer in a dilute solution (several volts ∼100k_B_T/e) that breaks the classic theory of electrokinetic phenomena [29].

**FIGURE 4:**
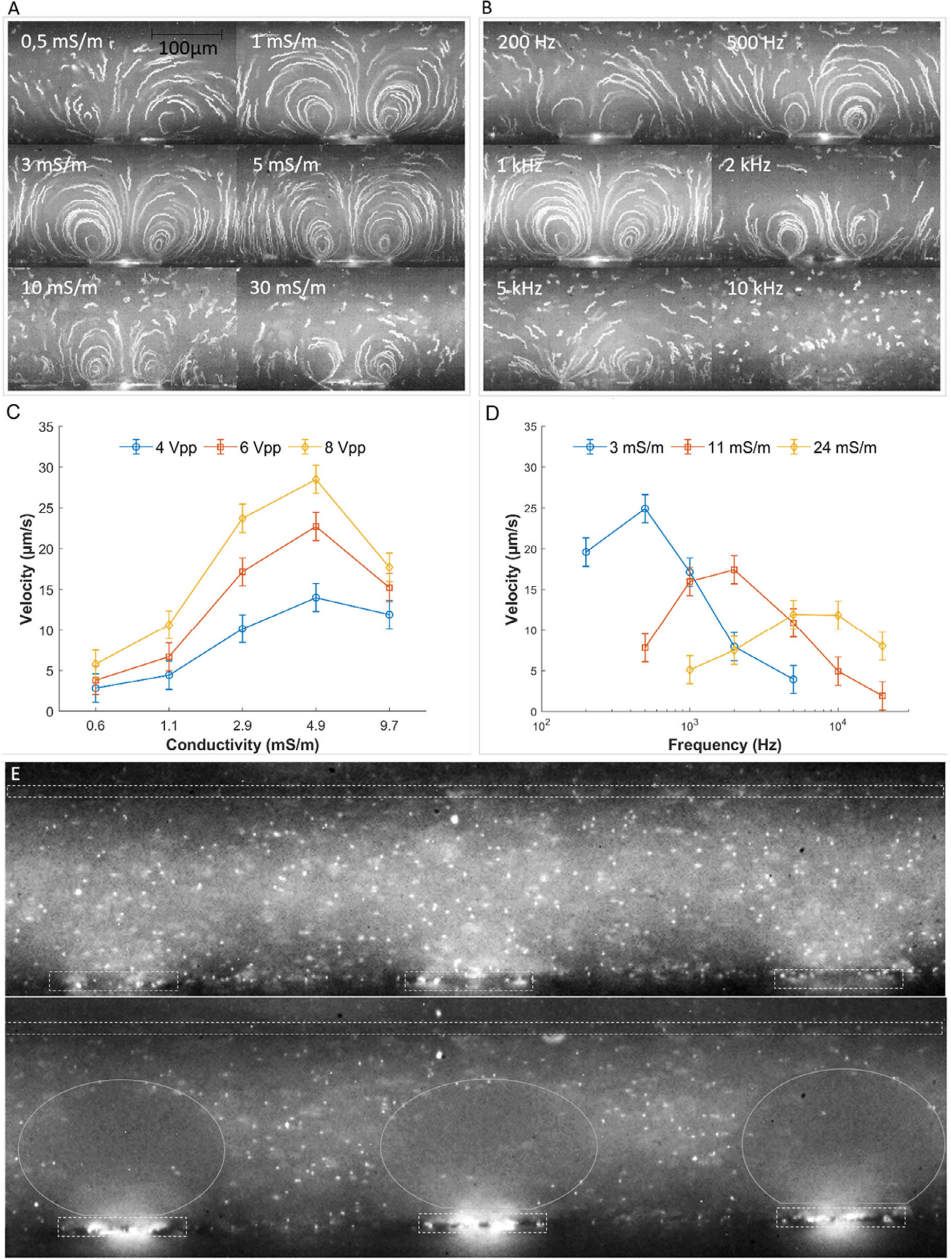
(A) Vortex images at fixed voltage (6 V_pp_) and fixed frequency (1 kHz); (B) vortex images at a fixed voltage (6 V_pp_) and fixed conductivity (3 mS/m); (C) plot of velocity as a function of conductivity for three voltages; (D) plot of velocity as a function of frequency for three conductivities. The error bars in the figures represent the uncertainty associated with the variability in manually selecting the fluorescent particle in ImageJ software across 10 measurements (type B uncertainty); (E) images of the microfluidic chamber before (top) and after (bottom) the application of the electric field for 30 s (*V* = 6 V_pp_, *f* = 1 kHz, $\;\sigma_{m}=1\;\mathrm{mS}/m$). The gray circles delimit the depletion zone and the grey rectangles outline the electrodes.

Experimental and numerical methods for determining slip velocity come with certain challenges. The slip velocity, measured horizontally along the electrode from the edge to the center, can be difficult to track accurately using the ImageJ plugin. This is primarily attributed to the tendency of fluorescent microparticles to become trapped on the electrode, potentially saturating the image in this region and complicating the analysis. As a result, the experimental measurements of electroosmotic velocity presented in Figure 4C,D were obtained manually by tracking fluorescent microparticles and calculating their velocity based on the time elapsed between images (1/60 s, to optimize contrast and tracking) and the pixel size.

Furthermore, although simulations offer valuable insights, the numerical model described in Section 3.6, which is based on COMSOL simulations, has opportunities for refinement. For instance, microparticles subjected to ACEO tend to adhere to the edge of the electrode. They may not be effectively driven toward the center due to the “stick” boundary condition applied to the electrodes on COMSOL. Furthermore, the model does not currently account for particle‐particle interactions, presenting a promising avenue for future improvement.

### 4.3 Experimental Study on Conductivity, Frequency, and Voltage Influence

The previously described device is a valuable tool for observing vortices, as shown in Figure 4A,B,E. Real‐time observation of vortex formation is obtained, and it allows for correlation with input parameters. After applying an electric field, images of the chamber show microparticles captured on the electrode, appearing as fluorescent spots. The brightest regions (Figure 4A,B,E), where the fluorescent microbeads are most concentrated, are located at the center of the electrodes, indicating that ACEO dominates over DEP. When the electroosmotic force weakens, then two fluorescent spots appear [11, 31] as illustrated at a frequency of 2 kHz (Figure 4B) and a conductivity of 0.5 or 1 mS/m (Figure 4A) and described in Figure 2D.

To facilitate comparison between input parameters, particle velocities (slip velocities near the electrode surface) were measured and plotted in Figure 4C,D.

As the voltage increases, the electric field intensifies, resulting in a higher electroosmotic velocity (Equation 4).

The study on varying conductivity (see Figure 4A) demonstrates that well‐defined vortices occur within the conductivity range of 1–5 mS/m for a 1 kHz frequency. Conductivities outside this range result in less defined or no vortices. As outlined in Section 2.2, at high conductivities the reduction in zeta potential leads to a decrease in electroosmotic velocity. In contrast, lower conductivities should result in a higher electroosmotic velocity, thereby improving fluid drag and enabling the collection of more microparticles on the electrode. However, when conductivity is too low, a decrease in electroosmotic velocity is observed. This can be attributed to the limited availability of ions, which restricts the generation of a significant electroosmotic flux. The applied electric field is unable to mobilize a sufficient number of ions to induce effective fluid movement, leading to a reduction in electroosmotic velocity. The primary driving force for electroosmotic flow—the product of the electric field and ionic charge density—is insufficient to overcome the fluid's viscosity [27].

With respect to frequency, well‐defined vortices are observed within the frequency range of 500–1 kHz, as discussed in Section 2.2.

Figure 4D illustrates that the optimal frequency for achieving higher velocity depends on conductivity and increases with it. Indeed, the optimal frequency for ACEO flow is closely tied to the conductivity of the medium. In low‐conductivity media, ions take longer to migrate and form the EDL due to their limited availability. As a result, lower AC frequencies are required to allow sufficient time for the EDL to charge and drive effective ACEO flow. In contrast, in high‐conductivity media, the EDL forms quickly because ions are abundant and migrate faster. Higher frequencies are needed in this case to ensure efficient energy transfer while avoiding parasitic effects such as electrolysis or excessive diffusion. This balance ensures optimal ACEO performance across varying conductivity levels. Conversely, as shown in Figure 4C, the medium conductivity required to achieve the highest velocity (5 mS/m in this study) remains constant across different applied voltages. A study on varying frequencies conducted by Green et al. [33] on coplanar electrodes shows similar trends in velocity behavior.

Additionally, this tool facilitates the differentiation between vortices induced by electroosmosis, characterized by known rotation direction (i.e., from the electrode edge to the center) and shape, and those resulting from ETEs or transitional phases between these phenomena, including instances of dual vortices [19, 30, 34].

Lastly, it allows for the observation of depletion zones (Figure 4E), where particles are carried away by vortices. This capability allows for studying how different parameters—such as conductivity, voltage, frequency, electrode size, and chamber height—influence the capture volume. Within seconds, particles present in the solution are captured on the electrode surface (see in the Supporting Information video), a feature particularly useful for improving the capture on surface‐based biosensors of biological objects such as cells or bacteria.

### 4.4 Numerical Study on the Influence of Chamber Geometry

This particle tracking method enables us to investigate how chamber geometry influences the capture of target elements. We examined the quantity of particles trapped on the electrodes as a function of the distance between them and the height of the chamber.

**FIGURE 5:**
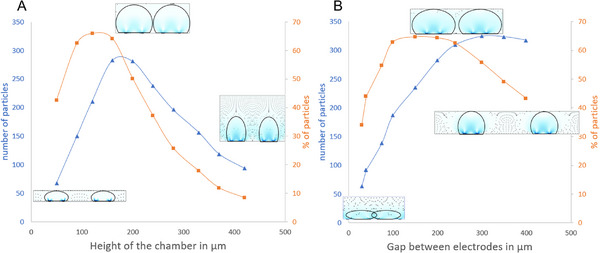
Number of 1 µm diameter microparticles trapped on the electrodes after 60 s is shown in blue, whereas the proportion of particles trapped on the electrodes is shown in red for (A) different inter‐electrode gaps (height = 160 µm) and (B) chamber heights (inter‐electrode gap = 100 µm). The electrical parameters are set at *f* = 1 kHz and *V* = 6 V_pp_, and the conductivity of the medium is 1 mS/m. The insets are not to scale.

In the study of geometric influence, as shown in Figure 5, the input parameters were set to a voltage of 6 V_pp_, a medium conductivity of 1 mS/m, and a correction factor of 0.02 applied in the simulation.

The number and proportion of microparticles trapped after 60 s were calculated for various geometries, as shown in Figure 5. The width of the electrodes was kept constant at 100 µm. The geometric parameters significantly influence the proportion of microparticles trapped on the electrodes. When varying the chamber height (from 50 to 420 µm), the inter‐electrode gap was fixed at 200 µm. Conversely, when varying the inter‐electrode gap (from 30 to 400 µm), the chamber height was fixed at 160 µm. The 1 µm microparticles were released at *t* = 0 s with a spacing in *x* and *y* transverse dimensions of 15 µm in a 1 mS/m medium.

Results show that a smaller inter‐electrode gap leads to a lower proportion of trapped microparticles, as the capture volumes induced by the vortices overlap. Conversely, if the inter‐electrode gap is too large, the capture volumes above each electrode fail to cover the entire chamber, resulting in a lower proportion of trapped microparticles. Similarly, if the chamber height is too large, the capture volumes cannot reach the entire chamber, again leading to a lower proportion of trapped microparticles. A small chamber height implies a smaller gap between the electrodes, which generates a stronger electric field and, thus, a faster electroosmotic velocity. However, the vortices become compressed and cannot carry a large proportion of particles.

As the particle density is constant in the simulation, the number of particles increases with the chamber size. The proportion of trapped particles (red plot) indicates that for a 100 µm comb electrode facing a flat gold electrode, the optimal spacing for trapping the maximum proportion of particles should be between 90 and 160 µm. For a 160 µm height, the comb electrode should have electrode spacing between 100 and 240 µm to achieve the best results.

## 5 Conclusions

A novel experimental tool for directly visualizing AC electrokinetic effects generated by top‐bottom electrodes is presented. To identify trends and to improve the understanding of ACEO phenomena with its dependence on parameters such as medium conductivity, applied frequency, and voltage, slip velocity was experimentally measured under various conditions. The effects of ACEO and DEP on latex beads were theoretically explained and consistently validated through experiments. Additionally, a numerical simulation, adjusted with an experimentally determined correction factor between 0.016 and 0.025, was performed using COMSOL to study the influence of electrode geometry. This numerical study demonstrates results similar to experimentation, both qualitatively (through the shape of vortices and the depletion zone) and quantitatively (through the velocity map).

Under the conditions of our study—1 µm latex microbeads in a microfluidic chamber between two top‐bottom electrodes spaced 160 µm apart—the optimal parameters for gathering the most microparticles on the electrode surface are a medium conductivity between 1 and 5 mS/m and a frequency between 500 and 1 kHz. These parameters are consistent with those reported in the literature for ACEO phenomena to dominate. This 2D numerical model and the approach outlined for selecting input parameters to concentrate targets on the surface can be easily adapted to various electrode and particle configurations tailored to the desired application.

Future work in the field of surface‐based biosensors could build on this study to enhance mass transport for biosensor applications by trapping micrometers of biological targets on the sensor area in a few seconds.

## Supporting Information

Additionally, photos, drawings, and schematics are provided to enhance understanding. This file also contains the process of obtaining key input values, such as latex microparticle conductivity, useful for COMSOL simulations.

## Supporting Information Videos

Experimental and Numerical video of vortices that trap microparticles on the electrodes.

## Conflicts of Interest

The authors declare no conflicts of interest.

## Supporting information



Supporting Information

Supporting Information

Supporting Information

## Data Availability

The data that support the findings of this study are openly available in Recherche Data Gouv at https://doi.org/10.57745/WMMBS6.
