# Supplementary material for: Electrohydrodynamic Vortex Imaging: A New Tool for Understanding Mass Transfer in Surface‐Based Biosensors
Source: Electrophoresis. 2025 May 10;46(17):1281–91. doi: 10.1002/elps.8137 (PMC12514429; doi:10.1002/elps.8137)
Supplement: Supplementary file 1 — Supporting Information [file ELPS-46--s002.docx]

**Supplementary** **file**

Pauline Zimmer^1,2*^, Oleh Andreiev^1,2,3,4^, Marion Costella^2,3,4^, Emmanuelle Laurenceau^1^, Jean-François Bryche^3,4^, Jean-Pierre Cloarec^1^, Michael Canva^3,4^, Marie Frénéa-Robin^2*^, Julien Marchalot^2*^

*Correspondence

P. Zimmer, M. Frénéa-Robin and J. Marchalot, Department of Bioengineering, Ampere Laboratory, Central College of Lyon, 69130 Ecully

E-mail : [pauline.zimmer@ec-lyon.fr](mailto:pauline.zimmer@ec-lyon.fr)

[marie.robin@univ-lyon1.fr](mailto:marie.robin@univ-lyon1.fr)

[julien.marchalot@insa-lyon.fr](mailto:julien.marchalot@insa-lyon.fr)

^1^Ecole Centrale de Lyon, INSA Lyon, CNRS, Université Claude Bernard Lyon 1, CPE Lyon, INL, UMR5270, 69130 Ecully, France

^2^Ecole Centrale de Lyon, Université Claude Bernard Lyon 1, INSA Lyon, CNRS, Ampère UMR5005, 69130 Ecully, France

^3^Laboratoire Nanotechnologies Nanosystèmes (LN2) -IRL3463, CNRS, Université de Sherbrooke, INSA Lyon, École Centrale de Lyon, Université Grenoble Alpes, Sherbrooke, J1K 0A5 Québec, Canada^4^Institut Interdisciplinaire d′Innovation Technologique (3IT), Université de Sherbrooke, 3000 Boulevard de l'université, Sherbrooke, J1K OA5 Québec, Canada

#
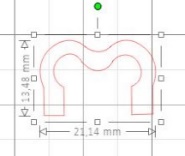


**Figure 1:** Drawing of the silicon seal forming the fluidic chamber of the device


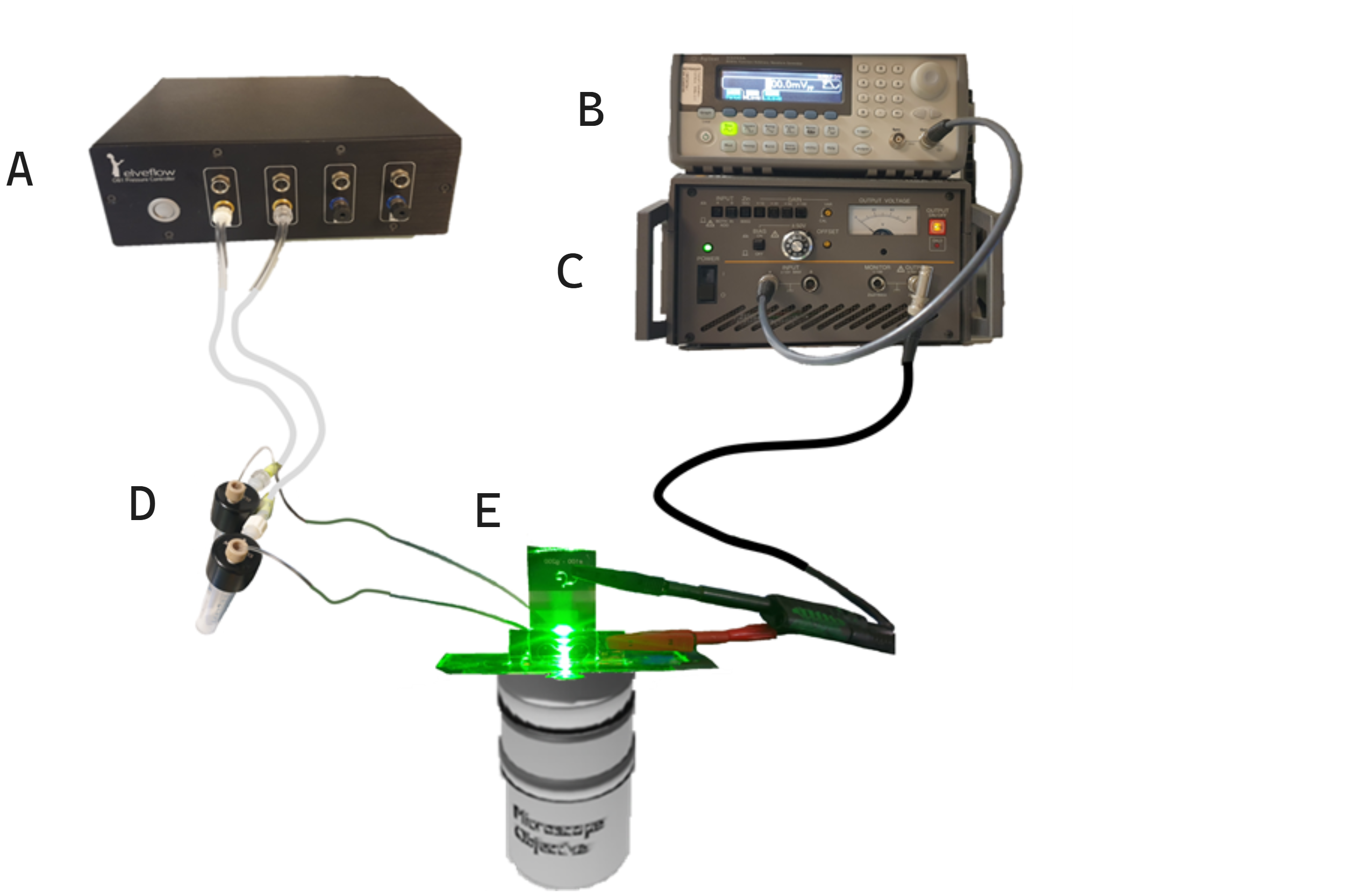


**Figure 2:** Photo of the experimental set-up: a) Microfluidic flow controller b) Voltage generator c) Amplifier d) Falcon^TM^ tubes for sample and waste e) Device for fluid flow visualization


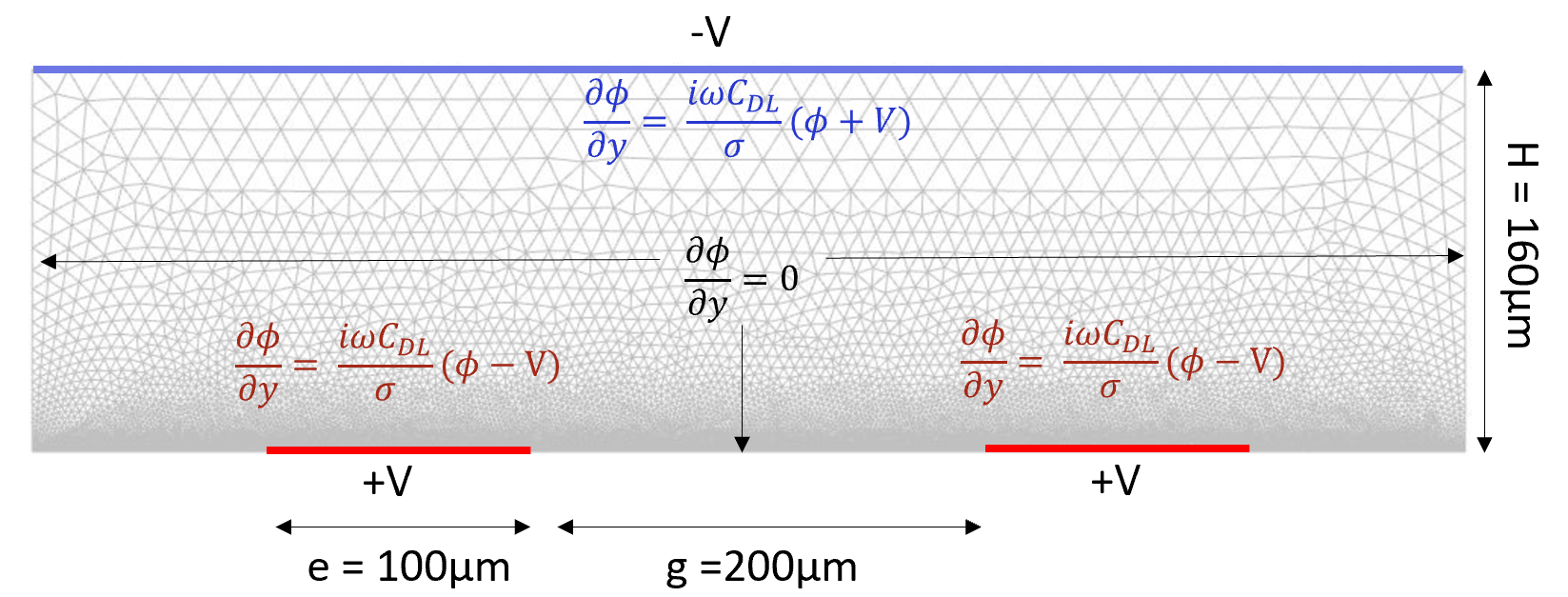


**Figure 3:** Robin-type boundary conditions, applied in the COMSOL Laplace equation module, for face-to-face electrodes


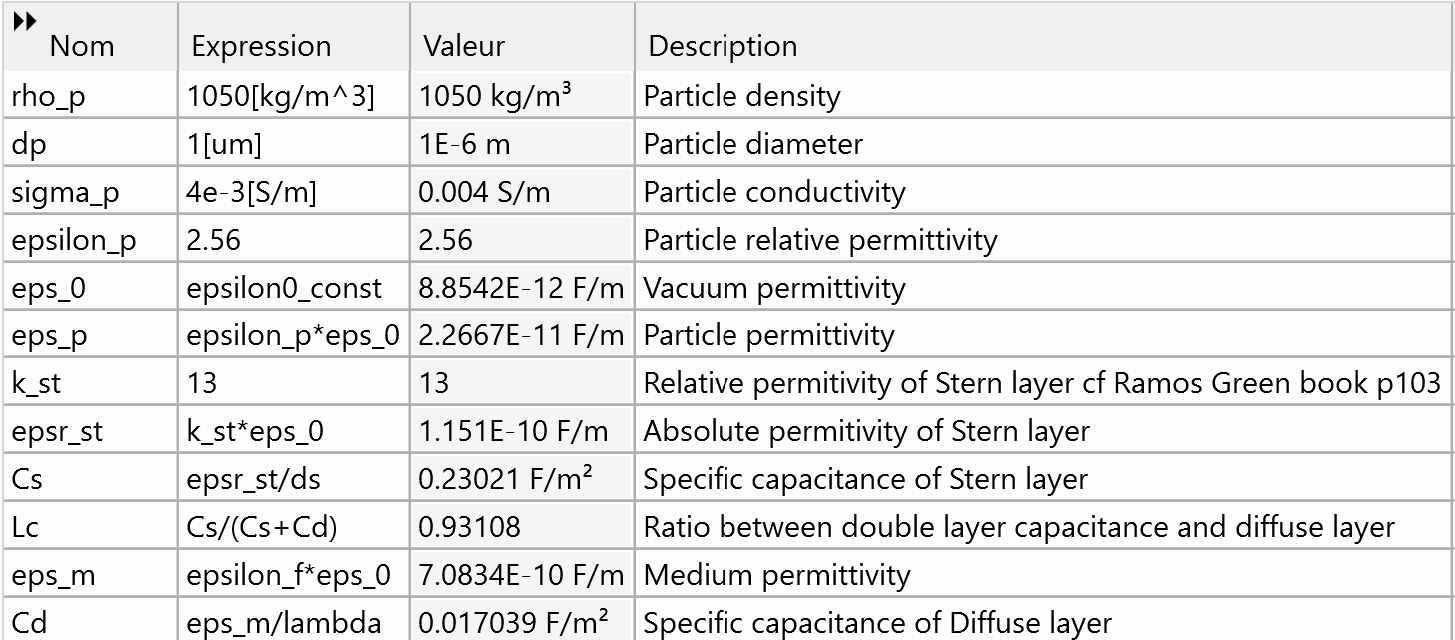


**Table 1 :** Latex beads properties and double layer capacitance values in COMSOL®

# Particle conductivity

The relative permittivity of latex beads is 2.56 [1].

The conductivity value of latex beads is required for the COMSOL® simulation, and this is how it was estimated:

It can be expressed as:

|  | $\sigma_{p}= \sigma_{pbulk}+\frac{{2K}_{S}}{r}$ | Equation 1 |
| --- | --- | --- |

Where $r$ is the particle radius, and $K_{S}$ the surface conductance, defined as $K_{S}={K_{S}}^{i}+{K_{S}}^{d}$, which represent the surface conductance of the Stern layer and the Diffuse layer, respectively [2].

Diffuse layer is expressed as follows:

|  | ${K_{S}}^{d}=\frac{4F^{2}cz^{2}D\left( 1+\frac{3m}{z^{2}} \right)}{RT\kappa}(\cosh\left[ \frac{zq\xi}{2\kappa T}-1 \right])$ | | Equation 2 |
| --- | --- | --- | --- |
|  |  |  | |

Where $F$ is the Faraday constant, $q$ is the electron charge, $c$ is the electrolyte concentration expressed in ${mol.m}^{-3}$, $z$ is the valency of the counterion, $D$ is the ion diffusion coefficient of, R is the molar gas constant, T is the temperature, $\kappa$ is the inverse Debye length and $m$is a dimensionless parameter describing the electroosmotic contribution to ${K_{S}}^{d}$, expressed as follows $m= {\frac{RT}{F}}^{2}\frac{2\epsilon_{m}}{3\eta D}$.

As the bulk conductivity ($\sigma_{pbulk}$) is negligible, the conductivity of latex beads depends solely on charges present on their surface.

The crossover frequency - at which the dielectrophoresis force is zero - allows for the calculation of $\sigma_{p}$. At this frequency, $Re(f_{cm}) = 0$, and knowing the dielectric properties of the medium in which the experiment was conducted, it is possible to determine the particle’s conductivity which depends only on its surface conductance. It means that the particle conductivity should be no longer valid if the medium is changed.

Experimentally, we found a crossover frequency around 550 and 700 kHz for 1 µm diameter particles in deionized (DI) water (measured at 0,1mS/m), which is consistent with the literature [3, 4]. After calculating the real part of the Clausius-Mossotti factor, the particle conductivity was found to be between 3.4 mS/m and 4.3 mS/m. In deionized (DI) water, the diffuse layer conductance is negligible compared to the Stern layer. The surface conductance is denoted as $K_{S}={K_{S}}^{i}$, and the conductance of the Stern layer can be considered constant relative to its value in DI water.

Moreover, in the range of medium conductivities used for the study (0.5 mS/m and 40 mS/m), the zeta potential, measured with a zeta sizer (Malvern), ranged between -33 mV and -44 mV +/- 4 mV, respectively and did not significantly change the value of ${K_{S}}^{d}$ (Equation 2) and thus $K_{S}$.

Therefore, we can consider that, in the conductivity range of the study, $\sigma_{p}$ remains approximately constant at 4 mS/m.

At low frequencies (below MHz), the real part of the Clausius-Mossotti factor approaches zero when the conductivities of the particle and the medium are equal, resulting in a negligible dielectrophoresis force. A positive dielectrophoresis force will occur when the medium conductivity is below that of the particles (less than 4 mS/m), while a negative dielectrophoresis force will arise when the medium conductivity exceeds that of the particles (greater than 4 mS/m).

Furthermore, the calculation of the electro-osmotic velocity requires knowledge of the capacitances of the Diffuse and Stern layer. The diffuse layer capacitance per unit area can be estimated from $C_{d}=\varepsilon_{m}\kappa$ [5] where $\varepsilon_{m}$ is the permittivity of the medium and 𝜅 is the inverse of the Debye length. The Stern layer specific capacitance is given by $C_{S}=\frac{\varepsilon_{s}}{d_{s}}$ and according to H. Morgan and N.G. Green [6] the Stern layer length $d_{s}$ is typically 0.5 nm and $\varepsilon_{s}$ is between 6 and 20.

# Drag Force

In fluid dynamics, the Reynolds number (Re) is a dimensionless quantity used to predict fluid flow patterns by measuring the ratio between inertial and viscous forces. It is defined as follows:

|  | $Re=\frac{\rho vd}{\mu}$ | Equation 3 |
| --- | --- | --- |

With ρ density of fluid in kg/m^3^, $v$ the velocity of the fluid in m/s, $d$ the characteristics length of solid in which is flowing in m and $\mu$ the dynamic viscosity of fluid in Pa*s. For velocity in the range of tens of µm/s the reynolds number is around 10^-5^. At such low reynolds numbers, viscous forces dominate the flow, an particle motion is governed by the balance between DEP force (expressed in Equation 1, section 2.1) and the drag force, expressed as follows for a spherical particle:

|  | $F_{drag}=6\pi\mu rv$ | Equation 4 |
| --- | --- | --- |

With r the radius of the spherical particle in m.

**References**

[1] Menad S, El-Gaddar A, Haddour N, et al. From Bipolar to Quadrupolar Electrode Structures: An Application of Bond-Detach Lithography for Dielectrophoretic Particle Assembly. *Langmuir* 2014; 30: 5686–5693.

[2] Hughes MP, Morgan H, Flynn MF. The Dielectrophoretic Behavior of Submicron Latex Spheres: Influence of Surface Conductance. *Journal of Colloid and Interface Science* 1999; 220: 454–457.

[3] Honegger T, Peyrade D. Comprehensive analysis of alternating current electrokinetics induced motion of colloidal particles in a three-dimensional microfluidic chip. *Journal of Applied Physics* 2013; 113: 194702.

[4] Wei M-T, Junio J, Ou-Yang HD. Direct measurements of the frequency-dependent dielectrophoresis force. *Biomicrofluidics* 2009; 3: 012003.

[5] Gagnon Z, Chang H-C. Aligning fast alternating current electroosmotic flow fields and characteristic frequencies with dielectrophoretic traps to achieve rapid bacteria detection. *Electrophoresis* 2005; 26: 3725–3737.

[6] Morgan H, Green NG. *AC electrokinetics: colloids and nanoparticles*. Baldock: Research Studies Press, 2003.
