# Supplementary figures and images for: Electrohydrodynamic Vortex Imaging: A New Tool for Understanding Mass Transfer in Surface‐Based Biosensors
Source: Electrophoresis. 2025 May 10;46(17):1281–91. doi: 10.1002/elps.8137 (PMC12514429; doi:10.1002/elps.8137)

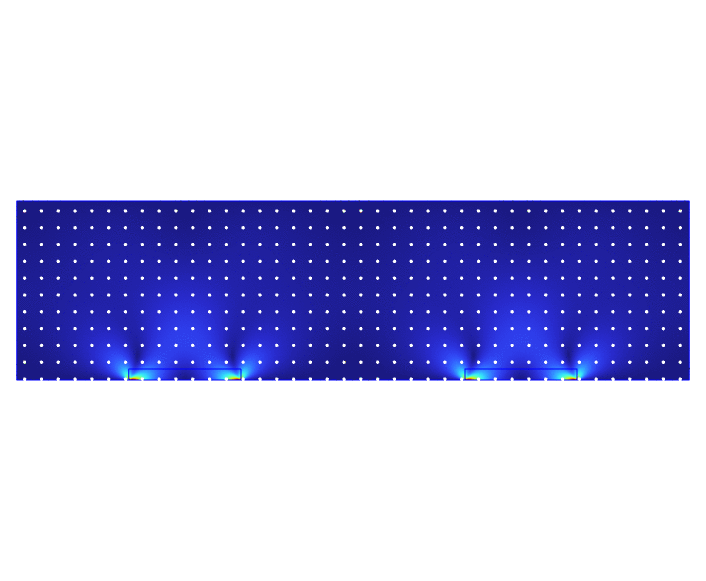

Supplement: Supplementary file 2 — Supporting Information [file ELPS-46--s001.gif]
